# Telmisartan Attenuates Uric Acid-Induced Epithelial-Mesenchymal Transition in Renal Tubular Cells

**DOI:** 10.1155/2019/3851718

**Published:** 2019-03-12

**Authors:** Dongqing Zha, Saiqun Wu, Ping Gao, Xiaoyan Wu

**Affiliations:** Division of Nephrology, Zhongnan Hospital of Wuhan University, Wuhan, Hubei 430070, China

## Abstract

We examined whether and how uric acid induces epithelial to mesenchymal transition (EMT) in renal tubular cells, along with the mechanism by which telmisartan acts on uric acid-induced renal injury. Rat renal proximal tubular epithelial cells (NRK-52E) were exposed to various concentrations of uric acid in the presence or absence of telmisartan. Treatment with uric acid increased the expression of *α*-SMA, decreased the expression of E-cadherin, and promoted EMT in NRK-52E cells. Uric acid treatment also led to increased endothelin-1 (ET-1) production, activation of extracellular-regulated protein kinase 1/2 (ERK1/2), and the upregulation of nicotinamide adenine dinucleotide phosphate oxidase 4 (NOX4). Use of ET-1 receptor inhibitor (BQ123 or BQ788) could inhibit uric acid-induced EMT in NRK-52E cells. Pretreatment with the ERK inhibitor (U0126 or PD98059) suppressed the release of ET-1 and EMT induced by uric acid. Additionally, pretreatment with a traditional antioxidant (diphenylene iodonium or apocynin) inhibited the activation of ERK1/2, release of ET-1, and uric acid-induced EMT in NRK-52E cells. These findings suggested that uric acid-induced EMT in renal tubular epithelial cells occurs through NADPH oxidase-mediated ERK1/2 activation and the subsequent release of ET-1. Furthermore, telmisartan (10^2^ nmol/L to 10^4^ nmol/L) inhibited the expression of NOX4, intracellular reactive oxygen species (ROS), activation of ERK1/2, and the release of ET-1 in a dose-dependent manner, thereby preventing uric acid-induced EMT in NRK-52E. In conclusion, telmisartan could ameliorate uric acid-induced EMT in NRK-52E cells likely through inhibition of the NADPH oxidase/ERK1/2/ET-1 pathway.

## 1. Introduction

Uric acid is the end product of purine metabolism. Excessive production of uric acid and decreased renal excretion leads to hyperuricemia, which is a risk factor for the development of chronic kidney disease [1]. Recent studies have shown evidence implicating a unique role of uric acid in the initiation and progression of renal fibrosis, which is a final common characteristic in chronic kidney disease [2].

Epithelial to mesenchymal transition (EMT) is a physiological or physiopathological process in which epithelial cells acquire the motile characteristics of mesenchymal cells [3]. EMT plays a prominent role in the accumulation of myofibroblasts and the resultant production of collagen and extracellular matrix, which is a critical step in the progression of renal fibrosis [4]. EMT is characterized by the acquisition of a fibroblast-like cell morphology, tight junction disruption, which is accompanied by decreased expression of epithelial markers, such as E-cadherin, and increased expressions of mesenchymal markers, such as *α*-smooth muscle actin (*α*-SMA), vimentin, fibronectin, and SM22 [5]. Recent evidence has shown that renal tubular epithelial cells undergo EMT phenotypic transformation in the development of uric acid-induced renal fibrosis [6, 7]. However, there is limited information regarding the role of uric acid in renal tubular epithelial cell injury and its underlying mechanisms.

High uric acid levels promote apoptosis in renal tubule cells by oxidative stress and activation of nicotinamide adenine dinucleotide phosphate (NADPH) oxidase 4 (NOX4) [8]. NOX4 has been implicated in the basal production of reactive oxygen species (ROS) in the kidneys, and its upregulation may promote renal oxidative stress and renal fibrosis [9]. There is evidence that high uric acid levels stimulate the production of NADPH oxidase-originated ROS, resulting in mesangial cell proliferation through the activation of extracellular-regulated protein kinase 1/2 (ERK1/2) pathway [10]. Elevated serum levels of endothelin-1 (ET-1) have been found in rats with hyperuricemia [11]. ET-1, an endogenous vasoactive factor, binds with endothelin receptors to exert its biological effects, including the regulation of renal blood flow, glomerular hemodynamics, and the urinary excretion of sodium and water [12]. ET-1 has two independent receptors in mammals, including type A and B, which are expressed on the surface of renal tubular epithelial cells [13, 14]. Accumulating evidence has shown that oxidative stress-induced ET-1 is involved in the EMT process of renal tubular epithelial cells and the resultant renal fibrosis [13]. Currently, is it unknown whether uric acid induces renal injury through the NADPH oxidase/ERK1/2/ET-1 pathway.

Telmisartan, an angiotensin II receptor blocker, is indicated for the treatment of hypertension [16]. Angiotensin II induces the activation of NADPH oxidase and oxidative stress, resulting in renal tubular injury [17]. A number of studies have shown that telmisartan ameliorates oxidative stress and prevents renal fibrosis in mouse models of diabetic nephropathy or unilateral ureteral obstruction [18–20]. High serum uric acid has been demonstrated to induce oxidative stress in renal tubular epithelial cells [8]. Telmisartan has been shown to reduce serum levels of uric acid independent of its antihypertension effects [21]. Despite possessing antioxidant potential, the beneficial effects of telmisartan against uric acid-induced renal injury have yet been elucidated.

Hence, we hypothesized that uric acid induced EMT in renal tubular cells through NADPH oxidase/ERK1/2/ET-1 pathway, while telmisartan could alleviate renal injury induced by uric acid. In this study, we examined whether and how uric acid induces EMT in renal tubular cells by examining the NADPH oxidase/ERK1/2/ET-1 pathway, along with the mechanisms by which telmisartan acts on uric acid-induced renal injury.

## 2. Materials and Methods

### 2.1. Cell Culture Reagents

Dulbecco's Modified Eagle medium (DMEM) and penicillin and streptomycin were purchased from HyClone (Logan, UT, USA). 10% fetal bovine serum (FBS) was obtained from Sijiqing Biological Engineering Materials Co. (Hangzhou, China). Uric acid, telmisartan, diphenylene iodonium (DPI), and ET(B) receptor antagonist BQ788 were purchased from Sigma (St. Louis, MO, USA). Apocynin, PD98059, UO126, and ET(A) receptor antagonist BQ123 were purchased from Selleck Chemicals (Houston, TX, USA).

### 2.2. Cell Culture and Treatments

The rat renal proximal tubular epithelial cell line (Normal Rat Kidney, NRK-52E) was purchased from the China Center of Type Culture Collection (Wuhan, China). NRK-52E cells were cultured in DMEM supplemented with 10% FBS, 100 IU/mL penicillin, and 0.1 mg/mL streptomycin at 37°C with 5% CO_2_. Cells were serum starved for 12 h, then incubated for 48 h at varied concentrations of uric acid (UA, 0, 100, 200, 400, and 600 *μ*mol/L). Cells were preincubated with the following agents for 2 h prior to subjected to uric acid (600 *μ*mol/L) for 48 h: telmisartan (10^2^ nmol/L to 10^4^ nmol/L) [22]; classic antioxidant DPI (20 *μ*mol/L) or apocynin (10 *μ*mol/L); ERK1/2 pathway inhibitor PD98059 (10 *μ*mol/L) or UO126 (10 *μ*mol/L); or ET-1 receptor inhibitor BQ123 (1 *μ*mol/L) or BQ788 (1 *μ*mol/L).

### 2.3. Assay of ET-1 Peptide Secretion

Cells were seeded in six-well plates and cultured under control and experimental conditions. The ET-1 concentration in the supernatant was measured using an enzyme linked immunosorbent assay (ELISA) kit (Elabscience Biotechnology Co.,Ltd, Wuhan, China) according to the manufacturer's protocol. The optical density, which was proportional to the ET-1 concentration, was determined spectrophotometrically at a wavelength of 450 nm ± 2 nm. Calculation of ET-1 concentration was accomplished using standard curves.

### 2.4. ROS Assay

Cells were plated and incubated onto glass coverslips in a six-well plate. After washing with phosphate buffered solution (PBS) for 5 min, the cells were then incubated with 5 *μ*M dihydroethidium (DHE) at 37°C for 30 min. The reaction mixture was aspirated, and the cells were washed with PBS for 5 min. 4′,6-Diamidino-2-Phenylindole (DAPI) counterstain was then applied to visualize nuclei. The antifluorescence quenching sealant was used, followed by observation and photography under a fluorescence microscope.

### 2.5. Quantitative Real-Time Polymerase Chain Reaction (PCR)

Total RNA was extracted using the Trizol reagent kit (Invitrogen Life Technologies, USA). The first strand complementary DNA (cDNA) was synthesized using the high-capacity cDNA reverse transcription kits (TaKaRa, Japan) according to the manufacturer's protocols. Real-time PCR was performed on an Applied Biosystems 7300 Real-time PCR System using SYBR green PCR reagent kits (TaKaRa, Japan). The primer sequences used are listed in Table 1, and all primers were obtained from Invitrogen Biotechnology. The relative amount of mRNA of each sample was calculated using the 2^−ΔΔCT^ method.

### 2.6. Western Blot Analysis

Anti-E-cadherin, anti-NOX4 and anti-GAPDH were purchased from Abcam (Cambridge, UK). Anti-pERK and anti-ERK were obtained from Cell Signaling Technology (Danvers, MA, USA). Anti-*α*-SMA was obtained from Tiandeyue (Beijing, China). Antifibronectin was purchased from Novus Biologicals (Littleton, CO, USA). Horseradish peroxidase (HRP)-conjugated secondary antibodies was obtained from SeraCare Life Sciences (Milford, MA, USA).

Cells were lysed in ice-cold radioimmunoprecipitation assay (RIPA) buffer (Beyotime, Shanghai, China) supplemented with proteinase inhibitor (Aspen Biotechnology, Wuhan, China) for 30 min. The lysates were centrifuged at 14,000 rpm for 10 min at 4°C. The protein concentrations were quantified using a bicinchoninic acid (BCA) protein assay kit (Beyotime, Shanghai, China). Subsequently, the protein lysates were denatured by boiling in sodium dodecyl sulfate (SDS) sample buffer at 100°C for 5 min. Protein samples were separated by 10% SDS-polyacrylamide gels and transferred to nitrocellulose (NC) membranes at 200 mA for 1.5 h at 4°C. After blocking with 5% nonfat milk, the membranes were incubated overnight at 4°C with primary antibodies (anti-P-ERK 1:1000; anti-ERK 1:1500; anti-*α*-SMA 1:5000; anti-E-Cadherin 1:1000; anti-fibronectin (1:500); anti-GADPH 1:10000; anti-NOX4 1:2000) and followed by incubated with HRP-conjugated secondary antibodies (1:10000) for 1 h at room temperature. The blots were visualized with an enhanced chemiluminescence (ECL) detection system (Boster, Wuhan, China). The density of each band was quantified with Image J software.

### 2.7. Immunofluorescence

The cell climbing film was fixed in 4% paraformaldehyde with 0.1% Triton X-100 for 30 min at room temperature. After blocking with 5% bovine serum albumin (Roche, Mannheim, Germany), films were incubated overnight at 4°C with primary antibodies (rabbit anti-E-cadherin 1:100; mouse anti-*α*-SMA 1:100). Then the cells were incubated with CY3-conjugated anti-rabbit IgG (1:50) or CY3-conjugated anti-mouse IgG for 30 min at room temperature. The immunolabeled cells were analyzed by fluorescence microscopy.

### 2.8. Statistical Analysis

The SPSS version 17.0 software package (IBM, Chicago, IL, USA) was used for statistical data analysis. All data were expressed as mean ± standard deviation (SD). Intergroup differences were analyzed by one-way analysis of variance (ANOVA).* P*<0.05 was considered statistically significant.

## 3. Results

### 3.1. Uric Acid Induced EMT in NRK-52E Cells

**Figure 1:**
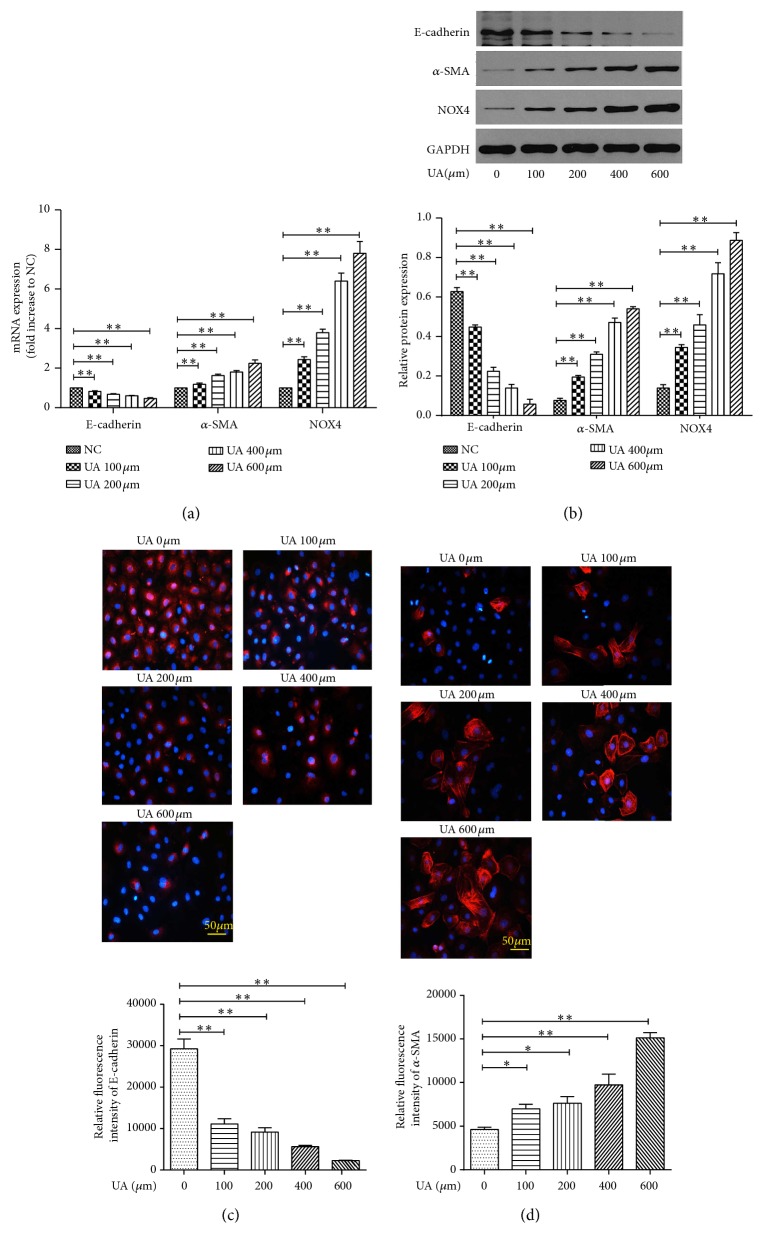
Effects of uric acid on EMT in NRK-52E cells. Cells were treated by different concentrations of UA (100-600 *μ*mol/L) for 48 h. (a) mRNA expression of NOX4, E-cadherin, and *α*-SMA by RT-PCR. (b) Protein expression of NOX4, E-cadherin, and *α*-SMA by Western blot. Immunofluorescence analysis of E-cadherin (c) and *α*-SMA protein (d). Magnification, ×200. The results are presented as mean ± SD obtained from three independent experiments. ^*∗*^*P*<0.05; ^*∗∗*^*P*<0.01. NC, normal control; UA, uric acid.

Exposure to uric acid (100 *μ*mol/L to 600 *μ*mol/L)* in vitro* led to a dose-dependently increased expression of the mesenchymal marker *α*-SMA, while expression of the epithelial marker E-cadherin was reduced in NRK-52E cells, at both mRNA and protein levels (Figures 1(a)–1(d)).

### 3.2. ET-1 Might Be Involved in Uric Acid-Induced EMT in NRK-52E Cells

**Figure 2:**
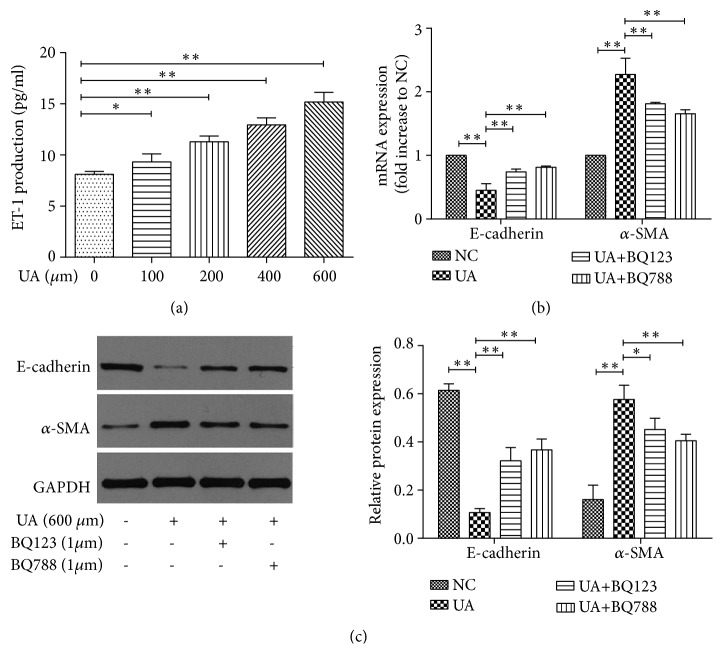
Role of ET-1 in uric acid-induced EMT in NRK-52E cells. (a) ET-1 production determined by ELISA when exposed to different concentrations of UA. Cells were treated by UA (600 *μ*mol/L) with or without ET-1 receptor inhibitor BQ123 (1 *μ*mol/L) or BQ788 (1 *μ*mol/L). E-cadherin and *α*-SMA expression were detected by RT-PCR (b) and Western blot (c). The results are presented as mean ± SD obtained from three independent experiments. ^*∗*^*P*<0.05; ^*∗∗*^*P*<0.01. NC, normal control; UA, uric acid.

Uric acid significantly increased the release of ET-1 in a dose-dependent manner (Figure 2(a)). Preincubation with the ET-1 receptor inhibitor, BQ788 or BQ123, could significantly reverse the increase of *α*-SMA expression and decrease of E-cadherin expression induced by uric acid, suggesting the involvement of ET-1 in the uric acid-induced EMT (Figures 2(b) and 2(c)).

### 3.3. Uric Acid Induced EMT in NRK-52E Cells through ERK1/2-ET-1

**Figure 3:**
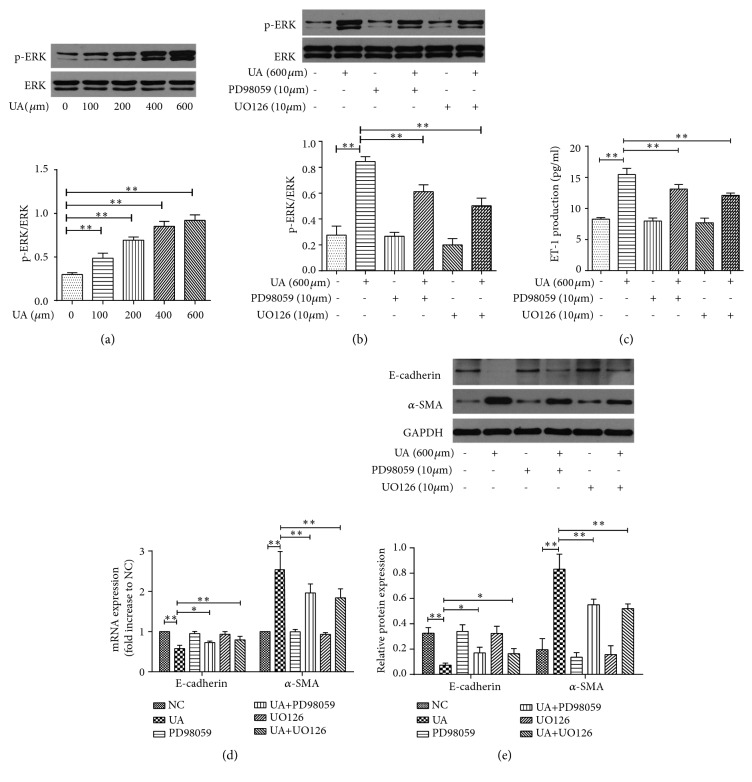
Role of ERK1/2 in uric acid-induced EMT in NRK-52E cells. (a) Protein expression of pERK and ERK by Western blot when exposed to different concentrations of UA. (b–e) Cells were treated by 600 *μ*mol/L UA with or without ERK1/2 pathway inhibitor PD98059 (10 *μ*mol/L) or UO126 (10 *μ*mol/L). Protein expression of pERK and ERK was detected by Western blot (b). ET-1 concentration in supernatant was measured by ELISA (c). mRNA expression of E-cadherin and *α*-SMA by RT-PCR (d). Protein expression of E-cadherin and *α*-SMA by Western blot (e). The results are presented as mean ± SD obtained from three independent experiments. ^*∗*^*P*<0.05; ^*∗∗*^*P*<0.01. NC, normal control; UA, uric acid.

Uric acid dose-dependently induced the activation of ERK1/2 in NRK-52E cells (Figure 3(a)). The ERK1/2 pathway inhibitor, PD98059, or UO126, could significantly inhibit uric acid-induced the activation of ERK1/2 (Figure 3(b)). Also, in the presence of uric acid, preincubation with PD98059 or UO126 could prevent the release of ET-1 (Figure 3(c)) and decrease the expression of *α*-SMA, while increasing E-cadherin expression, at both mRNA and protein levels (Figures 3(d) and 3(e)). These findings suggest that uric acid induced EMT in NRK-52E likely through the activation of ERK1/2 and promotion of downstream release of ET-1.

### 3.4. Uric Acid Induced EMT in NRK-52E Cells through NADPH Oxidase/ERK1/2/ET-1

**Figure 4:**
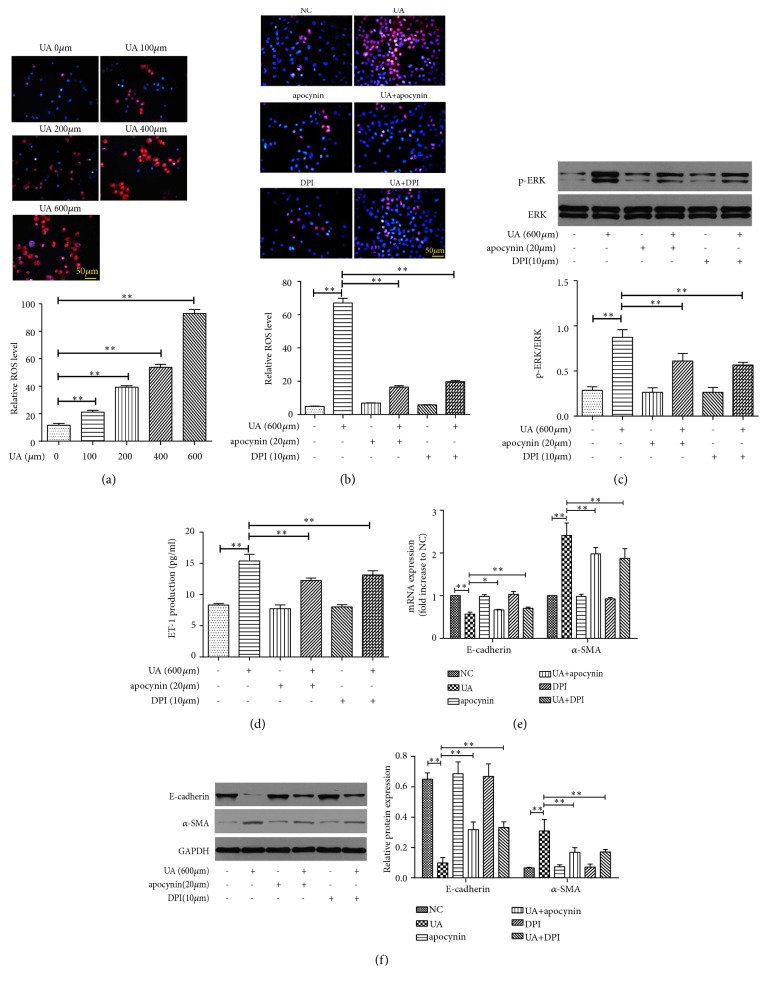
Role of NOX4/ROS in uric acid-induced EMT in NRK-52E cells. (a) ROS production measured by DHE staining when exposed to different concentrations of UA. Magnification, ×200. (b–f) Cells were treated by 600 *μ*mol/L UA with or without classic antioxidant apocynin (20 *μ*mol/L) or DPI (10 *μ*mol/L). Level of ROS determined by DHE staining. Magnification, ×200 (b). Protein expression of pERK and ERK by Western blot (c). ET-1 concentration in supernatant by ELISA (d). mRNA expression of E-cadherin and *α*-SMA (e). Protein expression of E-cadherin and *α*-SMA (f). The results are presented as mean ± SD obtained from three independent experiments. ^*∗*^*P*<0.05; ^*∗∗*^*P*<0.01. NC, normal control; UA, uric acid; DHE, dihydroethidium; ELISA, enzyme linked immunosorbent assay.

Intracellular ROS levels were detected to investigate the effect of oxidation on EMT induced by high uric acid. The results showed that uric acid increased the intracellular ROS levels in NRK-52E cells (Figure 4(a)).

NADPH oxidase enzymes are an important source of intracellular ROS and can be activated by uric acid in renal proximal tubule cells [8]. Thus, we further examined the expression of NOX4, a ROS-generating NADPH oxidase enzyme, in NRK-52E cells treated with uric acid. As expected, uric acid increased the mRNA and protein expressions of NOX4 remarkably (Figures 1(a) and 1(b)). Pretreatment with DPI and apocynin, two common antioxidants, could reduce uric acid-induced intracellular ROS formation (Figure 4(b)). Moreover, DPI and apocynin treatment significantly diminished the activation of ERK1/2 (Figure 4(c)), inhibited the production of ET-1 (Figure 4(d)), reduced the expression of *α*-SMA, and promote the expression of E-cadherin (Figures 4(e) and 4(f)) in NRK-52E cells treated with high uric acid. These results suggest a connection between ROS generation, activation of ERK, and production of ET-1, which together are implicated in uric acid-induced EMT in NRK-52E cells.

### 3.5. Telmisartan Inhibited Uric Acid-Induced EMT in NRK-52E Cells by Inhibiting the NADPH Oxidase/ERK1/2/ET-1 Pathway

**Figure 5:**
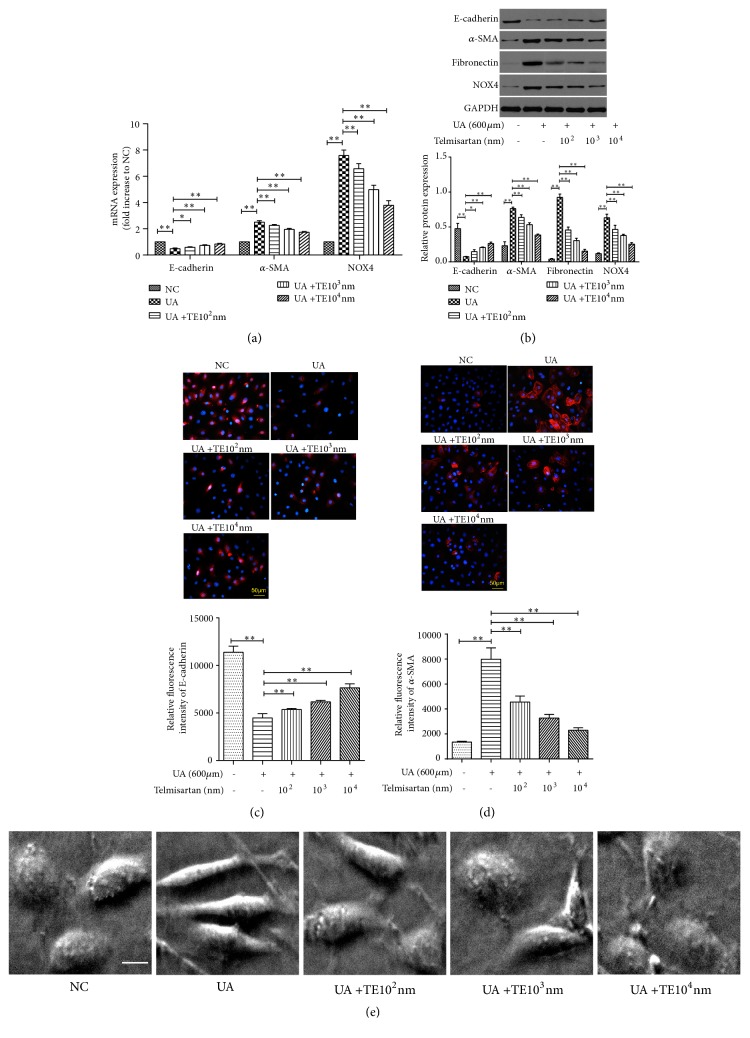
Telmisartan prevented uric acid-induced EMT in NRK-52E cells. Cells were treated by 600 *μ*mol/L UA with or without telmisartan (10^2^–10^4^ nmol/L) for 48 h. (a) Expression of NOX4, E-cadherin, and *α*-SMA mRNA levels by RT-PCR. (b) Expression of NOX4, E-cadherin, *α*-SMA, and fibronectin protein levels by Western blot. Expression of E-cadherin (c) and *α*-SMA (d) protein level and cellular localization by immunofluorescence. Magnification, ×200. (e) The morphological changes of NRK-52E cells by microscopy in different groups. Scale bar = 20 *μ*m. The results are presented as mean ± SD obtained from three independent experiments. ^*∗*^*P*<0.05; ^*∗∗*^*P*<0.01. NC, normal control; UA, uric acid; TE, telmisartan.

Pretreatment with telmisartan (10^2^ – 10^4^ nmol/L) could partially reverse the abnormalities in mRNA and protein expressions of E-cadherin, *α*-SMA and fibronectin induced by uric acid in NRK-52E cells, and these effects appeared to occur in a dose-dependent manner (Figures 5(a)–5(d)). Morphologically, the NRK-52E cells were arranged on the bottom of the culture flask, similar in appearance to paving stones. Under uric acid stimulation, the cells demonstrated an elongated and spindle-shaped appearance. After treatment with telmisartan, the morphologically aberrant cells reverted to a primarily normal appearance (Figure 5(e)).

**Figure 6:**
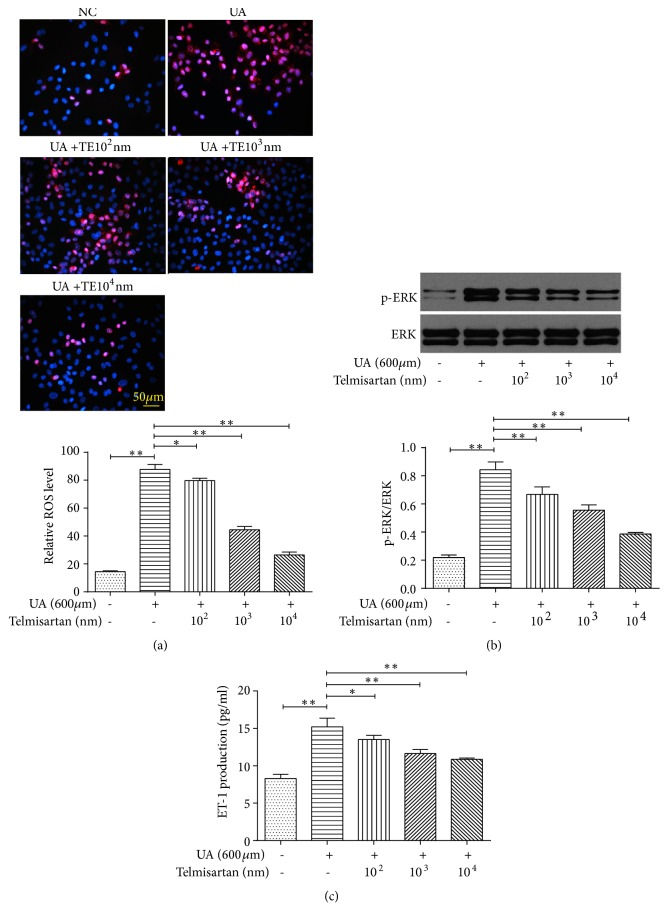
*Telmisartan inhibited uric acid-induced EMT in NRK-52E cells via inhibiting the NADPH oxidase/ERK1/2/ET-1 pathway*. Cells were treated by 600 *μ*mol/L UA with or without telmisartan (10^2^ – 10^4^ nmol/L) for 48 h. (a) Level of ROS was measured by DHE staining, Magnification, ×200. (b) Expression of pERK and ERK by Western blot. (c) ET-1 concentration in supernatant by ELISA. The results are presented as mean ± SD obtained from three independent experiments. ^*∗*^*P*<0.05; ^*∗∗*^*P*<0.01. NC, normal control; UA, uric acid; TE, telmisartan; DHE, dihydroethidium.

**Figure 7:**
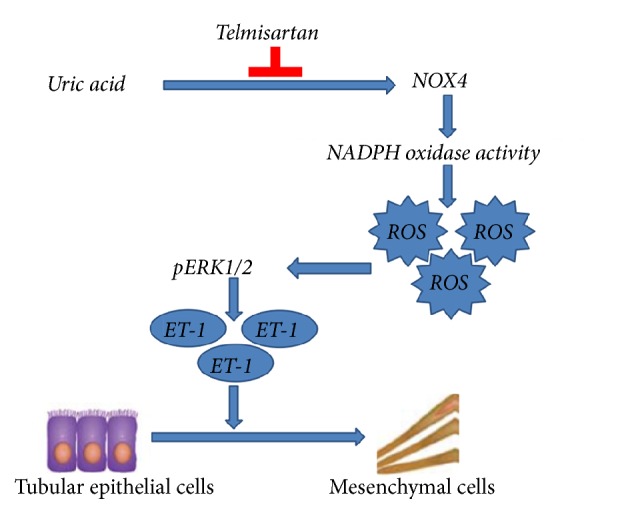
Schematic diagram of pathways involved in the protective effects of telmisartan on the inhibition of uric acid-induced EMT in NRK-52E cells [15].

Telmisartan significantly reduced uric acid-induced intracellular ROS formation (Figure 6(a)) and inhibited the expression of NOX4 (Figures 5(a) and 5(b)). Moreover, telmisartan treatment impeded the activation of ERK1/2 (Figure 6(b)) and prevented the production of ET-1 (Figure 6(c)). Taken together, these data suggest that telmisartan displays a protective effect against uric acid-induced EMT, likely by inhibiting NADPH oxidase and subsequent ERK1/2 activation and ET-1 production (Figure 7).

## 4. Discussion

This study showed experimental evidence suggesting that uric acid-induced EMT in renal tubular epithelial cells through a series of complex biological events, including NADPH oxidase-mediated ERK1/2 activation and subsequent release of ET-1. Importantly, telmisartan could significantly alleviate uric acid-induced EMT in renal tubular epithelial cells, as it prevented the changes in phenotypic markers. This protective effect of telmisartan might be linked with its capacity to decrease NADPH oxidase activity, reduce ROS production, as well as inhibit the release of ET-1.

Uric acid nephropathy is a disease caused by hyperuricemia-induced declines in kidney function. The deposition of uric acid in the renal interstitium can recruit inflammatory cells and cause chronic interstitial inflammation and fibrosis [23]. Recent studies have shown that uric acid could induce EMT in renal tubular epithelial cells [6, 7]. In this study, we found that treatment with uric acid led to altered molecular markers of EMT, including the upregulation of *α*-SMA and downregulation of E-cadherin.

Telmisartan, an angiotensin II receptor antagonist, may lower blood pressure, increase insulin sensitivity, inhibit the production of aldosterone, and protect renal function [24]. Telmisartan was also found to alleviate cardiac remodeling of dilated cardiomyopathy and retard or ameliorate organ fibrosis in diabetic mice [20, 25, 26]. Recently, telmisartan was reported to lower serum uric acid levels [21]. In our study, telmisartan partially reversed abnormalities in the EMT phenotypes (E-cadherin, *α*-SMA, and fibronectin) induced by uric acid, which was accompanied by morphological improvements in the NRK-52E cells. These findings suggest that telmisartan has a protective effect against uric acid-induced EMT in renal tubular cells.

ET-1 is involved in the process of myocardial and pulmonary fibrosis [27–29]. The levels were increased in the kidneys of rats with diabetic nephropathy or unilateral ureteral obstruction, which promoted renal interstitial fibrosis [30, 31]. In another study, serum ET-1 was increased following the establishment of a hyperuricemia rat model [11]. ET-1 may increase collagen accumulation in renal mesangial cells [32], and promote proliferation, inflammation, and ultimately renal fibrosis [13]. In this study, uric acid induced the production of ET-1, accompanied by acquisition of EMT phenotypes in renal tubular epithelial cells. Interestingly, telmisartan and ET-1 receptor antagonist (BQ788 or BQ123) could partly prevent uric acid-induced EMT and inhibit ET-1 production.

High uric acid levels can promote oxidative stress and reactive oxygen species production in renal tubular epithelial cells, resulting in a series of pathological changes including cell apoptosis and interstitial fibrosis [8]. NOX4, a member of NADPH oxidases, plays an important role in the progression of organ fibrosis, such as liver fibrosis, pulmonary fibrosis, and scleroderma [33–35]. NOX4 is highly expressed in the tubular cell compartment, and its upregulation may promote renal oxidative stress and renal fibrosis [36, 37]. NOX4 siRNA or NOX1/4 inhibitor (gkt136901) was previously found to prevent high glucose-induced fibronectin deposition in the kidneys and attenuate albuminuria in diabetic mice, revealing the renal protective effects of NOX1/4 inhibitor as a promising approach for the treatment of diabetic nephropathy [38, 39]. In addition, another study demonstrated that upregulation of NOX4 induced by uric acid promoted ROS production and apoptosis in human proximal tubule cell lines [8]. In our study, uric acid enhanced NADPH oxidase activity and ROS production, promoted the release of ET-1 and accelerated EMT in renal tubular epithelial cells. However, preincubation with telmisartan or common antioxidants (DPI or apocynin) could prevent these alterations and attenuate EMT in renal tubular epithelial cells. Our results confirm the role of the NOX4/ROS/ET-1 pathway and elucidate the mechanism by which telmisartan protects the kidneys against uric acid-induced EMT.

The excessive generation of ROS in the kidney can activate multiple intracellular signaling pathways that initiate the expression and transcription of the genes responsible for cell proliferation, eventually leading to excessive deposition of extracellular matrix and formation of renal fibrosis [40]. Previously, Cheng* et al.* found that uric acid increased NADPH oxidase activity, ROS formation, and ERK phosphorylation in cardiac fibroblasts [27]. Consistently, we found that uric acid treatment increased the ROS levels and activated ERK1/2 phosphorylation in renal tubular epithelial cells. However, telmisartan could prevent uric acid-induced alterations in ERK1/2. Taken together, telmisartan could ameliorate uric acid-induced EMT in NRK-52E cells, likely by inhibiting NADPH oxidase and subsequent ERK1/2 activation and ET-1 production (Figuer 7) [15].

In conclusion, our results demonstrated that uric acid induced EMT in renal tubular epithelial cells through the NADPH oxidase/ERK1/2/ET-1 pathway, which could be prevented or restored with telmisartan treatment. This study provided the experimental evidence supporting telmisartan as a promising agent for the treatment of uric acid-induced renal fibrosis.

## Figures and Tables

**Table 1:** Primer sequences used for real-time RT-PCR.

Genes	Primer sequences (5′-3′)
E-cadherin-F	TGCTTGAGAATGAGGTCGGTG
E-cadherin-R	TCAGAATGCCCTCGTTGGTC
*α*-SMA-F	CACCATCGGGAATGAACGCT
*α*-SMA-R	CTGTCAGCAATGCCTGGGTAC
NOX4-F	TGCATGTAGCTGCCCACTTG
NOX4-R	TTCAACAAGCCACCCGAAAC
GAPDH-F	CGCTAACATCAAATGGGGTG
GAPDH-R	TTGCTGACAATCTTGAGGGAG

## Data Availability

The data used to support the findings of this study are available from the corresponding author upon request.

## Abbreviations

EMT: Epithelial-mesenchymal transition
ET-1: Endothelin-1
PCR: Polymerase chain reaction
ROS: Reactive oxygen species.
